# Financial Sustainability and Corporate Social Responsibility Under Mediating Effect of Operational Self-Sustainability

**DOI:** 10.3389/fpsyg.2020.550029

**Published:** 2020-12-14

**Authors:** Rai Imtiaz Hussain, Shahid Bashir, Shahbaz Hussain

**Affiliations:** ^1^Department of Management Sciences, University of Okara, Okara, Pakistan; ^2^Business Studies Department, Namal Institute, Mianwali, Pakistan

**Keywords:** stakeholder theory, financial sustainability, corporate social (ir)responsibility, operational self-sustainability, microfinance

## Abstract

Operational and financial sustainability have, over time, remained as issues in the microfinance industry. The microfinance industry is struggling to gain self-sufficiency in Pakistan due to non-performing loans and operating costs. Simultaneously, deliberation on corporate social responsibility (CSR) is also considered in academic literature and organizational practices. However, studies on CSR and financial performance in the microfinance sector are scarce, especially in Pakistan. CSR will develop customer attraction and loyalty, employee attraction, motivation and commitment, MFIs' reputation and access to capital, and eventually build financial performance. Interviews were conducted with branch managers of microfinance institutions to test previous questionnaires. A self-administered survey was conducted to collect data from the managers of the microfinance banks operating in Punjab. Descriptive and inferential statistics were performed to answer research questions using Smart PLS. Most of the microfinance institutions believe in social responsibilities but lacks fund allocation and approval from higher management, and results are in line with prior studies. These empirical findings lead to the perception that CSR is not a barrier performance in microfinance banks as they have access to capital. The results indicated a strong positive correlation between CSR and the financial performance of the MFIs. CSR also positively correlates with customer retention, employees' motivation and attraction, and business reputation. CSR was associated with access to capital but was found to be weak. The research also narrated the limitation and practical implications of the study. The study also discusses further research directions.

## Introduction

The microfinance sector has gained attention in the last decade. Microfinance institutions (MFIs) provide microloans to the poor at their doorstep, which is costly and is the main hurdle in operational sustainability. Corporate social responsibility is a further added pressure on these MFIs to gain OSS and financial sustainability. Financial performance is the key to the future expansion of any enterprise. Financial sustainability (FS) is obtained through operational self-sustainability (OSS). It means that enterprises can only be financially sustainable if these are operationally economical (Hudon and Traca, 2011). Corporate social responsibility increases the share price and provides signals to prospective investors (Godfrey et al., 2009; Yang and Suvd, 2017; Hussain et al., 2020a,b). The debacle of corporate social responsibility (CSR) have been well-established in developed economies for the last three decades (Cochran and Wood, 1984; Torugsa et al., 2012), but remains a lively debate in emerging nations (Islam et al., 2017). A firm's CSR and financial performance are widely tested, but researchers do not agree on the same points, in terms of these variables' association. Prior studies were conducted to review the relationship between CSR and FS but no conclusive evidence could be found, nor could a consensus be reached on the nature of the relationship (Cochran and Wood, 1984; McWilliams and Siegel, 2000; Fauzi and Idris, 2009; Lin et al., 2009; Tang et al., 2012; Abdelkbir and Faiçal, 2015; Jiang and Yang, 2015; Akben Selcuk and Kiymaz, 2017).

Corporate social responsibility is still an essential issue for the microfinance sector as the social impact can only be achieved through outreach (Woller, 2007; Shu and Oney, 2014; Nurmakhanova et al., 2015; Cho et al., 2019). Targeting low income customers is the primary concern for large and sustainable MFIs (Thomas and Jyothi, 2016). Therefore, MFIs have to invest resources in social performance that distract MFIs from core objectives of profitability and operational and financial sustainability (Woller, 2007; Nurmakhanova et al., 2015; Naz et al., 2019). The target for financial sustainability puts pressure on the MFIs to distract CRS and to target easier-to-reach rich customers to reduce the default risk. This action will result in dislodging from a mission to provide services to the unbanked. On the other hand, CSR will increase social roots, customers' loyalty, poverty alleviation, employee attraction, and access to capital (Cochran and Wood, 1984; Woller, 2007; Sweeney, 2009; Naz et al., 2019). Thus, MFIs have to trade-off between CSR and financial self-sufficiency (Nurmakhanova et al., 2015).

The paper contributes to prior research in three areas. The mediating effect of operational self-sustainability is ignored, which is more significant in the microfinance industry as it has a higher operating cost than conventional banking (Naz et al., 2019). CSR in microfinance institutions (MFIs) is more important than any other industry as MFIs receive donations from donors to enhance outreach and social projects (Sweeney, 2009). Finally, CSR and financial performance are mostly tested through secondary data based on historical data (Abdelkbir and Faiçal, 2015; Manokaran et al., 2018). The current study used primary data obtained from the managers [consistent with prior studies of Sweeney (2009)] of the larger pool of MFIs to find the relationship between CSR and financial sustainability.

The research paper's sequence includes the theory and hypothesis development of financial sustainability, operational self-sustainability, and the corporate social responsibility of MFIs. The following passage discusses the material and data used in the analysis and the empirical findings generated in the study. Furthermore, results of previous reviews, limitations, and implications thereof are also discussed.

## Theory and Hypotheses Development

This study looks at the modern finance theory, i.e., efficient market hypothesis, signaling theory, reputation theory, and stakeholder theory to develop hypotheses. Stakeholder theory focuses not only on the interests of stockholders but also fulfills the CSR toward stakeholders, both internally and externally (Woller, 2007; Sayekti, 2015; Rhou et al., 2016; Freeman and Dmytriyev, 2017). Signaling theory posits signals to the company's interested users (Watts and Zimmerman, 1978; Godfrey et al., 2009). Thus, Watts and Zimmerman (1978) signaling theory urges one to follow full disclosure assumptions so that stakeholders have complete information about the enterprise. Modern finance theory, i.e., efficient market hypothesis (EMH), reflects that all information about the assets is readily available to the investors and is reflected in the share price (Malkiel, 2003, 2005; Fama and French, 2004). Therefore, the financial performance of the company will send the signal to investors for their future decisions.

### Financial Sustainability

Microfinance institutions can cover all expenses, operational costs, financial costs, and service expenses to enhance equity market value and to achieve their social goals (Thomas and Jyothi, 2016). MFIs charge a high-interest rate to attain financial sustainability, which is often criticized by the customers and policymakers in developing countries like Pakistan, India, and Bangladesh (Thomas and Jyothi, 2016). Return measures financial performance on Assets (Sweeney, 2009), which is the core goal of all stakeholders. Most prior studies (Cochran and Wood, 1984; Tucker, 2001; Hartarska and Nadolnyak, 2007; Sweeney, 2009; Gibson, 2012; Thomas and Jyothi, 2016) look at the return on equity (ROE), return on assets (ROA), return on sales, and earnings per share (EPS). These studies also find a positive association between CSR and financial performance. Financial sustainability is measured through two approaches; accounting returns and investor returns (Cochran and Wood, 1984; Lin et al., 2015).

#### Investor Returns

Prospective investors always want to know about returns on their investment. Investor return was employed in the studies of Moskowitz (1972) to measure the enterprise's financial performance and was then later used in many other studies (Cochran and Wood, 1984; Naz et al., 2019). Price per share was used as an investor return in Moskowitz (1972) studies, which was later found to be faulty. The dividend yield is also used to measure investor returns (Moskowitz, 1972; Cochran and Wood, 1984). These two measures of investor returns disregard the element of risk.

The finance theory or capital asset pricing model measures the risk and returns of holding assets (Cochran and Wood, 1984; Fama and French, 2004). The concept of “beta” is introduced, which is the slope of regression. The average coefficient is one, and if the stock beta is below 1, the stock is considered defensive, while if stock beta is over 1, it is considered aggressive (Cochran and Wood, 1984; Fama and French, 2004). Later, modern finance theory, the efficient market hypothesis, was generated, affecting future cash inflows and share prices (Fama, 1991; Fama and French, 2004; Hussain et al., 2020a,b).

#### Accounting Returns

Accounting returns remain the other measures for financial performance (Cochran and Wood, 1984). The advantage of using accounting returns is to see the enterprise's implementation of reporting standards and managerial policies. Accounting returns are based on historical data, which leads to inflation that is the drawback of these measures (Cochran and Wood, 1984). Three accounting returns are employed in the studies of Cochran and Wood (1984); (1) the ratio of EBIT to assets, (2) the ratio of EBIT to sales, and (3) excess market value. Cochran and Wood (1984) study discussed the specific weakness of financial leverage differences as firms are selected from different industries. The said issue does not arise in the present study as we have collected primary data from the managers of the MFIs.

Prior studies used earnings per share (ESP), return on equity (ROE), and return on assets (ROA) as a proxy for financial performance in accounting measures (Tsoutsoura, 2004; Wafula et al., 2017; Cho et al., 2019). The current study also employs ESP, ROE, and ROA as an accounting measure to test financial performance. Still, the information is gathered through a questionnaire from the managers of MFIs banks.

### Corporate Social Responsibility

Corporate social responsibility is the society's expectation from organization operating in their locality (Baldo, 2014; Sayekti, 2015; Galdeano et al., 2019). CSR is part of business ethics, and business ethics must be followed in corporate sectors (Christensen et al., 2007). Furthermore, the World Bank described that “companies with social responsibilities always think about their impact on environment, communities, and stakeholder goals to achieve profit.” Companies with CSR responsibilities have to think about customers, employees, the environment, and its reputation, which is known as win-win strategies (Sayekti, 2015; Tuan, 2016). Nicolopoulou (2011) highlights the prominence of knowledge transfer toward CSR literature which helps in understanding the concept better.

CSR has a positive impact on sales, share price, and profit, leading to financial performance (Yang and Suvd, 2017). Jaakson et al. (2009), Loew et al. (2004), and Galdeano et al. (2019) defined CSR as “a concept whereby companies integrate social and environmental concerns in their business operations and their interaction with their stakeholders” voluntarily. CSR includes social responsibilities like legal, economical, and ethical activities (Cho et al., 2019) and a firm's contribution toward society, but these are not followed adequately in developing countries (Ofori and Hinson, 2007).

CSR's role as a moderating variable is tested in the studies of Tuan (2016) on organizational ambidexterity-entrepreneurial orientation relationships. CSR has positively moderated the relationship between both variables. CSR activities are not performed in all industries that never served CSR activities, but claimed regular exercises as CSR activities (Cherapanukorn and Focken, 2014). Furthermore, SMEs and family firms cannot correctly implement social and environmental practices (Murillo and Lozano, 2006; Marques et al., 2014).

Social performance in the microfinance industry is the outreach of microfinance to low income customers which is the objective of microfinance institutions (Woller, 2007; Thomas and Jyothi, 2016). The activities covered under social performance in MFIs include targeting customers and assessing the customers' needs (Thomas and Jyothi, 2016). In the current study, social responsibility involves customer retention, employees' trust in their MFIs to perform in terms of social objectives, social acceptance, and social capital building (Sweeney, 2009). These objectives of social performance will increase the future sustainability of the enterprise. CSR is mostly applied in enterprises but is not tested in MFIs.

#### Customers Retention

Corporate social responsibility contributed positively toward the enterprise image and developed the customers' trust in the firms that had enhanced the organization's financial performance (Galdeano et al., 2019). Customer retention is a benefit of CSR activity in an organization, which eventually contributes to sales and profit (Lee and Heo, 2009; Lee and Shin, 2010). On the other hand, customers argue that firms actively involved in CSR activities are trusted and produce higher quality products (McWilliams and Siegel, 2000). Prior studies claim a positive impact of CSR on sustainability and that it also increases customer retention (Berman et al., 1999; Brammer and Pavalin, 2006; Brammer et al., 2007; Carmeli et al., 2007).

Consumers are more interested in the firm's CSR activities than traditional factors like product price, quality, intrinsic value, and the financial performance of the firm (Brammer and Millington, 2008; Sweeney, 2009; Jose et al., 2012). The evidence of consumers' interest in CSR can be reviewed in many prior studies ranging from theories, blogs, magazines, books, and publications like “Shopping for a Better World.” Sometimes, customers even care more about CSR activities than product quality and price (Sweeney, 2009). Prior studies mainly focus on customer retention as a formative construct of CSR in manufacturing firms but it is mostly ignored in the microfinance sector. Therefore, this gap is filled in the present study.

#### Employees Attraction and Loyalty

Corporate social responsibility also motivates internal employees (Skudiene and Auruskeviciene, 2012) to increase their commitment toward their work and firm (Brammer et al., 2007; Collier and Esterban, 2007). Employee engagement increases in firms where CSR activities are performed, and these activities impact the businesses in various positive ways (Hurst and Ihlen, 2018). Employees' loyalty develops toward multiple benefits like higher performance, improved customer service, and attracts new employees (Galdeano et al., 2019). It means that employees with higher loyalty and engagement put forward their best efforts to increase the financial performance of the organization (Brammer and Pavalin, 2006; Brammer et al., 2007; Brammer and Millington, 2008).

A potential applicant for a job prefers to apply to firms that are engaged in CSR activities. Furthermore, firms with CSR attract more applicants to open positions (Sweeney, 2009). The findings are furthered confirmed, in that potential employees pay closer attention to the firms' contribution to environmental issues, community projects, and diversity issues (Sweeney, 2009). Employee loyalty and attraction are considered in all manufacturing firms but is not used in the microfinance sector.

#### Enterprise Reputation

Enterprise reputation is an intangible asset and often deals with goodwill (Davies and Miles, 1998). Goodwill is sold and narrated in financial statements at different values using International Accounting Standards (IASs). This reputation affects the share value in the long-run and satisfies stakeholders' satisfaction with the firm's policies (Siano et al., 2010; Baldarelli and Gigli, 2014). Stakeholder theory and reputation theory are the drivers of corporate social responsibilities. The relationships of enterprise reputation and corporate social responsibilities in practice have already been tested in many prior studies and contribute to the literature. Reputation is an intricate marvel but is the primary formative variable of CSR (Janney and Gove, 2011).

CSR develops the enterprise's reputational capital, which increases public trust (Tang et al., 2012) and market value, indicating financial performance (Jiang and Yang, 2015; Yang and Suvd, 2017). CSR contributes to reputation theory and, in return, enhances corporate financial performance (Wang and Shenghua, 2016). Prior studies focused on the nature of the relationship between CSR and CFP in firms that had outperformed the market (Moskowitz, 1972, 1975; Galdeano et al., 2019). Likewise, CSR activities increase the enterprise's financial performance, which increases the enterprise itself (Brammer and Pavalin, 2006; Brammer et al., 2007; Brammer and Millington, 2008; Iamandi, 2012). Reputation was tested as a mediating variable in the studies of Sweeney (2009) between CSR-FP.

The resource-based view generates a competitive advantage and signals to shareholders and investors who want to make future contracts with the firm (Sweeney, 2009). Prior studies (Brammer and Pavalin, 2006; Sweeney, 2009; Siano et al., 2010) found a positive association between enterprise reputation and financial performance. Therefore, a firm's good reputation enhances share market values, and people trust the firm's information, whereas a lousy reputation reduces the market value of products and services. Therefore, the authors wanted to see the importance of MFIs' contribution in CSR activities.

#### Social Capital Availability

Under the resource-based view, the CSR-CFP link enhances the social capital for firms engaged in social and environmental activities (Brammer and Pavalin, 2006; Brammer et al., 2007; Brammer and Millington, 2008). More resources are allocated for CSR activities by some corporate companies. Some companies resisted the concept of additional investment in society for the environment and other activities as it reduces its profit (McWilliams and Siegel, 2000). Firms performing CSR activities have a greater chance of accessing social capital. Potential investors choose to invest in firms with adequate CSR (Baron, 2008). Sweeney (2009) also mentioned in his studies that creditors like credit unions, banks, and MFIs lean more toward firms with social responsibilities. Therefore, the authors wanted to test social capital available for firms with more CSR activities.

### Corporate Social Responsibility and Financial Performance

Corporate social responsibility and financial performance have been reviewed in many prior studies in both developed and developing economies, and mixed results have been found, therefore, a meta-analysis was conducted and is discussed in the following subheadings.

#### Developed Economies

Cochran and Wood (1984) provided evidence of a weak positive correlation among CSR and FS in 39 firms registered in America. Yang and Suvd (2017) analyzed CSR's impact on the financial performance of 16 low-cost airlines. CSR increases the financial performance of carriers. Wang and Shenghua (2016) reviewed CSR and CFP links in the meta-analytic framework in 42 studies. The relationship was found to be positive and significant and supported the stakeholder theory. CSR and CFP also support the market efficiency hypothesis. The association of CSR and financial performance is more notable for developed countries than in developing countries; however, it was found to be neutral in McWilliams and Siegel (2000) study.

Rhou et al. (2016) researched CSR awareness as a mediating variable on CSR and FP's association in 5,812 restaurants in Northern America from CPI. The results indicate that CSR awareness affects the initiatives of the managers for CSR and financial performance. The data for 500 companies registered in the American stock exchange from 1998 to 2008 were collected for the analysis of CSR and intellectual capital and financial performance was collected from the Compustat database (Lin et al., 2015). The results indicate a direct impact of CSR on FP through the mediating effect of intellectual capital. Tsoutsoura (2004) demonstrated a positive and significant impact of social responsibilities on financial performance in S&P 500 firms in Northern America.

The broader Canadian firms were motivated to issue separate CSR reports as they faced political and societal pressures. In contrast, small firms were found to be less-interested in the publication of information (Thorne et al., 2014). It generates concerns that even in developed countries, small firms hesitate to take part in CSR activities. Tang et al. (2012) collected longitudinal data from 130 firms of the S&P 500 from 1995 to 2007 to establish the CSR-CFP relationship in the presence of an engagement strategy. The results could not, however, establish the relationship of CSR-CFP.

Stubbs and Schapper (2011) worked on sustainability and CSR in the educational institutes of Australia. The authors' used a case study on two subjects of corporate sustainability. CSR and sustainability have a positive relationship. Australian SMEs were researched in Torugsa et al. (2012), where the authors empirically tested the association of proactive CSR and FP. The study results are consistent with the BRV theory and found its capabilities to improve financial performance. Sweeney (2009) used the structural equational model in SMEs and larger firms to determine CSR and FP's positive association and obtained results consistent with prior studies.

#### Emerging Markets and Developing Economies

Fauzi and Idris (2009) researched the association of CSR and corporate financial performance, of the good management theory and the slack resource theory of firms in Indonesia. The findings showed that CSR positively impacted the financial performance of companies. Sayekti (2015) studied Indonesia Stock Exchange companies for 4 years to determine the relationship between strategic CSR and non-strategic CSR and financial performance. The empirical findings showed a positive effect of strategic CSR on financial performance, whereas non-strategic CSR was negatively associated with FP.

The relationship between social performance and financial sustainability of MFIs in India was assessed by Thomas and Jyothi (2016). The financial sustainability of MFIs is different from conventional banks and are measured differently as it includes the balance between social and financial performance. Akben Selcuk and Kiymaz (2017) found a relationship between firm performance and CSR in firms listed in Borsa Istanbul and used the content analysis to obtain data from financial statements. The results showed a negative association among the variables.

A study was conducted by Cho et al. (2019) on 191 firms listed at the Korea stock exchange to measure CSR performance and financial performance (profitability, firm value). Profitability was measured through return on assets. The empirical evidence found a positive relationship between CSR performance and profitability and firm value. The association was also tested in the studies of Platonova et al. (2018), where the authors found a significant positive association of CSR disclosure and financial performance in the Islamic banks of GCC over 15 years.

Another study was conducted by Ofori and Hinson (2007) in Ghana to gain insight on CSR's perspective in 100 leading firms. The prior study was further extended in Kuada and Hinson (2012) studies in Ghana, where local firms adopt CSR policies according to society's local culture. Galdeano et al. (2019) predicted future financial performance through CSR and the moderator role of organizational engagement in Bahrain's banking industry. The findings showed a positive relationship of CSR on the FP and reported a significant impact of organizational engagement on the CSR-FP relationship. Doh et al. (2015) focused on the emergence of CSR and sustainability in Brazil's emerging markets. The authors worked on the impact of societal, institutional, and organizational (CSR activities) on society.

Using the extensive literature on the CSR and FP in both developed and developing countries, CSR was applied to the Aviation industry, Higher Education, Restaurant Industry, SMEs, Islamic banks, and manufacturing firms. The CSR and Financial performance were not tested in the microfinance sector of Pakistan. The following hypothesis was generated for testing.

**Hypothesis 1**: Corporate Social Responsibility is positively attached to Financial Sustainability.

Esampally and Joshi (2016) identified five OSS determinants in India's MFIs and non-banking financial companies. These include yield on GLP, total assets, cost per borrower, GLP, and several active borrowers. The findings showed that the increase of OSS could be obtained through a rise in total assets and yield on GLP, while OSS will decrease with cost per borrower and active borrowers in MFIs. Strategies were developed for CSR and sustainability in developing countries' multinational enterprises (DCMNES). CSR directly improves the OSS of the companies and enhances the firm's value (Doh et al., 2015). The following hypothesis can therefore be generated.

**Hypothesis 2**: Corporate Social Responsibility is positively associated with Operational Self-Sustainability in the microfinance sector of Pakistan.

#### Operational Self-Sustainability

The operational cost of MFIs is higher than other banks as these provide services to the unbanked at their doorstep (Naz et al., 2019). The MFIs have to perform this function to raise low-income customers' income levels (Akram and Hussain, 2011). Therefore, measurement of OSS (revenues minus operational expenses) is a better approach than FSS (Rai et al., 2010; Schäfer and Fukasawa, 2011; Rai and Rai, 2012). Operational self-sufficiency is expressed in percentage and shows whether MFI covers operating cost, financial cost, and loan losses, and is achieved if it is more than 100 percent (Esampally and Joshi, 2016). OSS can be found by reducing cost or increasing revenues (Adongo and Stork, 2006; Schäfer and Fukasawa, 2011; Beg, 2016).

The financial sustainability in MFIs can be obtained through the gaining of operational self-sufficiency in the long term, which reduces cost and increases efficiency (Adongo and Stork, 2006; Balkenhol, 2007; Rai et al., 2010; Rai and Rai, 2012; Hamad and Duman, 2013; Velnamby and Alagathurai, 2014; Balagobei, 2016; Beg, 2016; Esampally and Joshi, 2016; Lensink et al., 2018). Khan and Sulaiman (2015) reported the inefficiency of MFIs in operating cost and loan officers and optimal use of financial assets. It means that an MFI is financially sustainable if it is operationally self-sustained. The following hypothesis can therefore be generated.

**Hypothesis 3**: Operational Self-Sustainability is positively associated with financial sustainability.

Corporate Social Responsibility was directly tested with Financial performance in many prior studies (Adongo and Stork, 2006; Balkenhol, 2007; Rai et al., 2010; Rai and Rai, 2012; Hamad and Duman, 2013; Velnamby and Alagathurai, 2014; Balagobei, 2016; Beg, 2016; Esampally and Joshi, 2016; Lensink et al., 2018) but operational self-sustainability is the critical variable in the Microfinance sector. OSS's role cannot be ignored as the literature depicts that an MFI is financially viable if its operational cost is less than its operating income, whereas, CSR increases the operational expenses and decreases the profitability of the firm. The study therefore tests the following hypothesis.

**Hypothesis 4**: Operational Self-Sustainability mediates the relationship of CSR and Financial Sustainability in MFIs operating in Pakistan.

**Figure 1:**
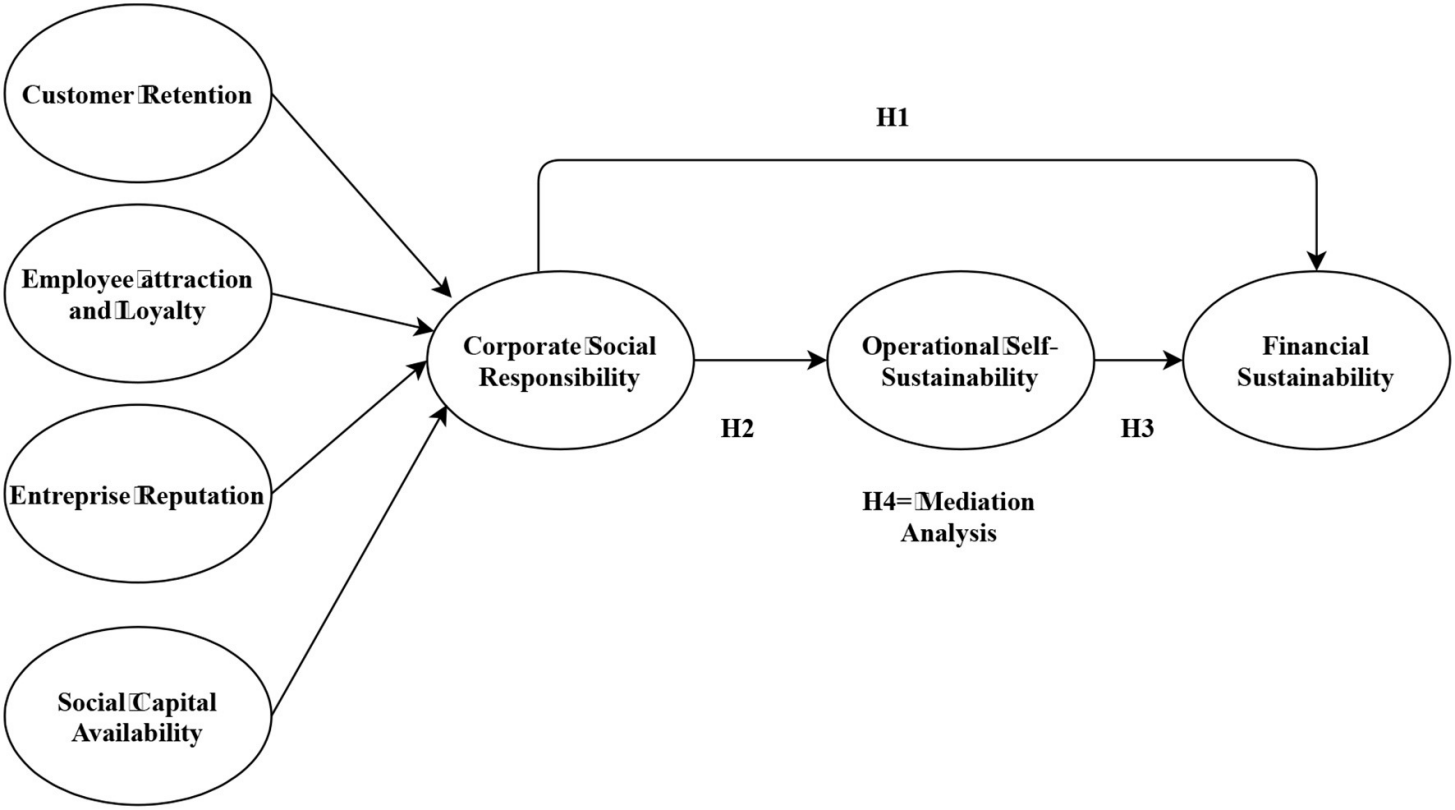
Theoretical framework.

Figure 1 depicts the theoretical framework, explaining corporate social responsibility as an independent variable, operational self-sustainability as a mediator, and financial sustainability as a dependent variable.

## Materials and Methods

### Data Collection and Analysis

Pilot testing was conducted with 30 Territory and Area Managers to validate the adaptation of the questionnaire of Sweeney (2009) along with rigorous testing before final the self-administration (Saunders et al., 2019). In the pilot testing, the acceptable response rate was achieved as the researcher personal visited the respondents' offices. Questions that were not adequately understood by mid-level managers, were modified again.

A common method, the survey method, was used to collect the data from the 1,400 branch managers of large MFIs operating in Pakistan. Seven hundred questionnaires were posted and emailed to managers in Sindh, Khyber Pakhtunkhwa and Balochistan, Gilgit Baltistan, and Azad Jammu Kashmir. In contrast, data from Punjab and Islamabad were personally collected. A judgmental sampling technique was used to collect data from managers of MFIs. The response rate for posted and emailed questionnaires was 31%, whereas personally administrated questionnaires obtained a response rate of over 69%. A total of 422 completed questionnaires were collected. Only 372 questionnaires were useable. The demographic information of the respondents is provided below in Table 1.

**Table 1:** Demographic information of respondents.

**Variables**	**Categories**	**Frequency**	**Percentage**
Gender	Male	233	62.6
	Female	139	37.4
Marital status	Single	194	52.15
	Married	178	47.85
Age	≤ 20	30	8.06
	21–25	166	44.6
	26–30	140	37.6
	≥31	36	9.74

### Measures of Variables

In prior studies, CSR has generally been measured in two ways (Cochran and Wood, 1984) (Table 2). First, it is calculated based on some indicators determined by experts in the relevant field of CSR. The second method has already been used in the studies of Moskowitz (1972, 1975), where the reputation index was developed with a ranking scale of “outstanding,” “honorable,” and “worst.” Both of these measures are more subjective. Therefore, other dimensions of CSR are used in this study which can easily measure the variable, i.e., customer retention (CR), employee attraction and loyalty (EL), enterprise reputation (ER), and access to capital (SC) (Cochran and Wood, 1984; Sweeney, 2009; Jose et al., 2012; Tang et al., 2012; Torugsa et al., 2012). A five-point Likert scale was used to measure these variables.

**Table 2:** Measurement of variables.

**Variable**	**Description**	**References**
Corporate social responsibility	CSR covers the social responsiveness performed by MFIs in the society, i.e., Customers Retention, Employees Attraction and Loyalty, Enterprise Reputation and Social capital access	Cochran and Wood, 1984; Marrewijk, 2003; Sweeney, 2009; Jose et al., 2012; Tang et al., 2012
Operational self-sustainability	Operational self-sufficiency is obtained if the cost of advancement is less than the revenue generated from lending/loans	Sweeney, 2009; Vanroose and D'Espallier, 2013; Daher and Saout, 2015; Doh et al., 2015; Naz et al., 2019
Financial sustainability/performance	Financial sustainability is calculated through Earnings per share, dividend yield, Return on Assets, Return on Investment, Net profit to sales, the stock price	Cochran and Wood, 1984; Sweeney, 2009; Tang et al., 2012; Torugsa et al., 2012; Sayekti, 2015; Thomas and Jyothi, 2016; Meyer, 2019

Compared to CSR, financial performance is challenging to measure as researchers have not reached a consensus on a measurement method. However, financial performance is measured through investors' returns and accounting returns (Cochran and Wood, 1984; Sweeney, 2009; Torugsa et al., 2012).

## Results

The data was collected through a questionnaire, and is known as primary data. To analyze the primary data gathered in the collection process, Smart PLS 3.0 is applied. This software has many advantages over others. Formative constructs can be interpreted as possible with the help of Smart PLS, whereas covariance-based software like AMOS cannot handle this. The present study applied PLS-SEM to analyze and validate the relationship between the defined variables in the model.

### Measurement Model

As shown in Table 3, SmartPLS tests the reliability and provides the values for Cronbach's alpha and composite reliability (CR) of all defined variables in the model. The values that are >0.70 are acceptable; thus, all values of the variables meet the requirements of CR cut off (Marakas et al., 2007). Both Cronbach's alpha and CR are used to calculate the reliability of the questionnaire. The Average Variance Extracted (AVE) is estimated to determine convergent validity. The convergent validity threshold criterion is that the AVE should be higher than 0.50, for all the build (Hair et al., 2016). The values suggested that these variables satisfy those requirements.

**Table 3:** Reliability analysis.

**Variables**	**Cronbach's alpha**	**Rho_A**	**Composite reliability**	**Average variance extracted (AVE)**
CR	0.935	0.936	0.949	0.756
EL	0.898	0.899	0.929	0.667
FS	0.891	0.904	0.924	0.753
OSS	0.931	0.945	0.948	0.785
Reputation	0.932	0.933	0.947	0.748
Social capital	0.918	0.921	0.939	0.754

The present study applied the well-known criteria (Fornell and Larcker, 1981). It describes that AVE's square root should be greater than its correlation with any other latent variables in a model. Table 4 explains that AVE's square roots are greater than the correlation of other latent variables, which confirmed the condition of discriminant validity.

**Table 4:** Discriminant validity.

**Variables**	**CR**	**EL**	**FSS**	**OSS**	**Reputation**	**Social capital**
CR	0.870					
**EL**	0.417	0.876				
**FS**	0.480	0.480	0.868			
OSS	0.523	0.510	0.457	0.886		
**Reputation**	0.600	0.524	0.501	0.527	0.865	
Social capital	0.541	0.438	0.528	0.513	0.624	0.868

*Significant at 0.05.

HTMT correlation ratio is also determined and was based and proposed by Henseler et al. (2015). It is a new instrument used for assessing discrimination's legitimacy. HTMT 's maximum appropriate value for verifying discriminant validity is 0.85, whereas any value above suggests a validity issue (Henseler et al., 2015). The findings of the HTMT are provided in Table 5.

**Table 5:** HTMT.

	**CR**	**EL**	**FS**	**OSS**	**Reputation**	**Social capital**
CR						
EL	0.616					
FS	0.326	0.421				
OSS	0.601	0.541	0.326			
Reputation	0.643	0.404	0.121	0.683		
Social capital	0.601	0.512	0.431	0.579	0.389	

### Formative Constructs

The present study applied the latest convictions (Hajli, 2014; Gaskin et al., 2018). Corporate social responsibility (CSR) is used as a multidimensional construct in this research, so it is essential to validate its four dimensions. After applying the guidelines suggested by Gaskin et al. (2018). The results proved that the four dimensions (Social capital, reputation, EL, and CR) are traits of corporate social responsibility and are shown in Table 6. These figures demonstrated that CSR could work as a higher-operative construct in this study.

**Table 6:** Validating formative constructs.

**Relationship**	**Type**	**Original mean**	**Standard deviation**	**T statistic**	***P*-value**
CR → CSR	1st → 2nd	0.294	0.007	39.944	0.000
EL → CSR	1st → 2nd	0.200	0.006	34.475	0.000
Reputation → CSR	1st → 2nd	0.293	0.008	35.037	0.000
Social capital → CSR	1st → 2nd	0.244	0.008	31.171	0.000

### Common Method Biased Variance

Data were obtained from a single source and is cross-sectional, so Harman's single-factor test was used to verify the common system variance (CMV). Since a popular method was used in data collection, spurious covariance shared among variables was tested (Podsakoff et al., 2003). An exploratory factor analysis of all the build products' items showed that the first two factors cumulatively account for 39.92% of the variance, with the first factor accounting for 33.52% and the second factor explaining 6.39% of the overall variance. The single factor did not account for any variance, which means the data was not influenced.

### Structural Model

**Figure 2:**
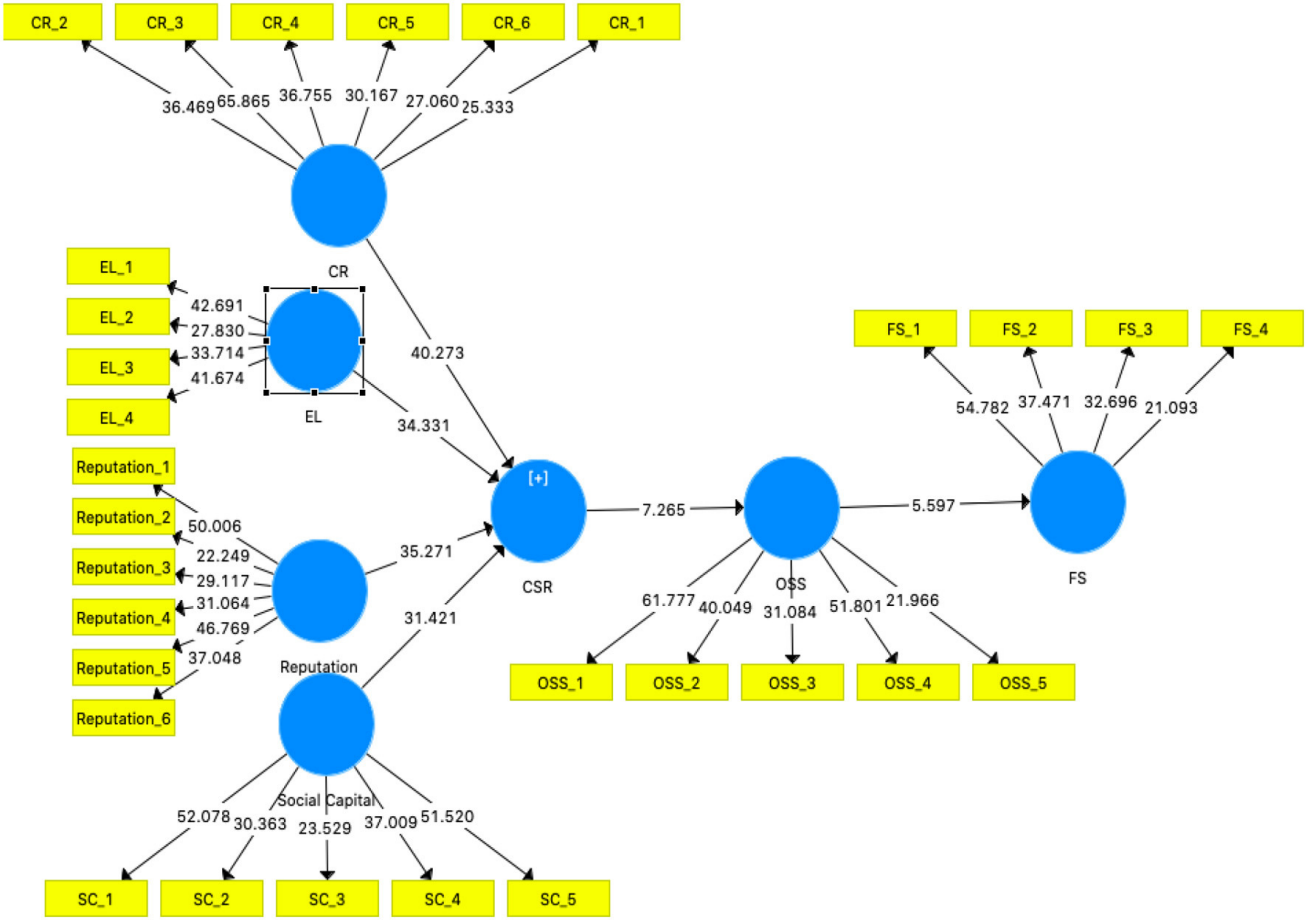
Structural model results.

The scores are calculated from Smart PLS which appear in Table 7 and Figure 2. As the results show, each relationship is significant and noteworthy at the 0.05 level. The model's validity is determined by R square estimation (Hair et al., 2010). R square has shown that 30.12% of the change in financial sustainability occurred due to operational self-sustainability and a 35.09% change in operational self-sustainability due to corporate social responsibility. For specific endogenous latent constructs, the Q^2^ values measured must be >0 in the SEM. It demonstrates that the *Q*^2^ values were equal to 0.401 and 0.311 for this study model, respectively, which was higher than the threshold limit, and supports the predictive relevance of the path model for the endogenous construct. The present study is deductive because it is used to clarify the relationships made in the model. The structural equation modeling technique was applied through bootstrapping and implemented to get the results of t-statistics. The bootstrapping of 5,000 resamples and 372 cases explained that corporate social responsibility significantly impacted operational self-sustainability, proving H1. Operational self-sustainability also has a significant and positive effect on financial sustainability (*T* = 5.59, *p* < 0.05). Furthermore, the present study has also validated the results of H3, proving that corporate social responsibility has a positive impact on financial sustainability (*T* = 3.90, *p* < 0.05). Table 7 and Figure 2 explains the results of the hypotheses explained in the research model.

**Table 7:** Hypothesis results.

**Relationship**	**Original mean**	**Standard deviation**	**T statistic**	***P*-value**	**Hypothesis supported?**
CSR → OSS	0.536	0.074	7.265	0.000	Yes
CSR → FS	0.245	0.063	3.905	0.001	Yes
OSS → FS	0.457	0.082	5.597	0.002	Yes

### Mediation Analysis

To test for the mediating role through H4, the present study engaged the latest conventions (MacKinnon et al., 2002; Hayes, 2013), focused on bootstrapping. For the mediating effect, the indirect effect must also be significant (Hussain et al., 2020). Operational self-stability means working as a mediator, mediating corporate social responsibility and financial sustainability. The present study analyzed and discovered that corporate social responsibility has a significant and positive relationship with financial sustainability. Furthermore, the indirect effects of the hypothesis were also substantial. Table 8 describes mediation results, and this hypothesis is partially mediated. It further shows that Variance accounted for (VAF=indirect effect/Total effect) 22.53% of operational self-sustainability.

**Table 8:** Mediation analysis.

**Relationship**	**Direct effect**	**Indirect effect**	**Total effect**	**VAF**	**Type of mediation**
CSR → FS	0.251				
CSR → OSS → FS		0.251*0.288 = 0.073	0.324	22.53%	Partial

## Discussion and Conclusion

The study employed SmartPLS to test the direct association between CSR and financial performance, and the formative construct of CSR activities: customer retention, employees' attraction and loyalty, social capital, and enterprise reputation. Furthermore, the mediating effect of operational self-sufficiency on the CSR and financial performance relationship was also tested.

The formative construct of CSR activities, CR, EL, Reputation, and social capital, were significant positive contributors toward CSR. Customer retention was deemed a vital benefit of CSR and ultimately increased sales and profitability. Customers were more interested in CSR activities in the prior studies (Berman et al., 1999; Brammer and Pavalin, 2006; Brammer and Millington, 2008). The findings were also consistent with Sweeney (2009) investigations in Ireland and Cochran and Wood (1984) in America, where weak positive association was found. Employee loyalty is another benefit of CSR that positively impacts its financial efficiency (Brammer et al., 2007; Collier and Esterban, 2007; Brammer and Millington, 2008; Sweeney, 2009). This study's results are consistent with prior reviews and found a positive association between employee loyalty and CSR.

Enterprise reputation is another dimension of CSR and found a positive association between reputation and CSR in prior studies (Brammer and Pavalin, 2006; Sweeney, 2009; Iamandi, 2012; Tang et al., 2012; Wang and Shenghua, 2016). The current study results indicate a positive contribution of a firm's reputation in CSR activities, consistent with prior studies. Social capital is more readily available for those firms which are engaged in CSR activities. People trust CSR-based firms and invest in those (McWilliams and Siegel, 2000; Brammer and Pavalin, 2006; Baron, 2008; Sweeney, 2009). Social capital was significantly positively correlated to CSR—consistent with the prior studies.

The prior literature review highlighted the issue of non-consensus on the definition of corporate social responsibility (Cochran and Wood, 1984; Christensen et al., 2007; Sweeney, 2009; Lee and Shin, 2010; Marques et al., 2014; Rhou et al., 2016; Naz et al., 2019). Each author presumes a different concept of CSR activities, and this was the first objective of this study—to see whether MFIs understand the CSR term in Pakistan. The common definition through the stakeholder theory was developed by narrating customers, community, environment, and employees. During the study, it was found that large MFIs were more familiar with the concept of CSR than small/new MFIs (Sweeney, 2009).

CSR is the topic of great importance in Pakistani culture (Khan and Sulaiman, 2015; Khan et al., 2017; Naz et al., 2019) and South Asian countries (Cassar and Wydick, 2010; Jose et al., 2012; Sim and Prabhu, 2014; Thomas and Jyothi, 2016). The results also proved that CSR activities are an essential topic for Pakistani society, consistent with prior studies.

Hypothesis 1 shows the association of CSR and financial self-sufficiency, which are studied in many prior studies both in developed and emerging economies. The results generated were contradictory, and researchers did not reach a consensus on the relationship. CSR had a weak positive correlation with financial performance in the studies of Cochran and Wood (1984), whereas a strong positive association was found in the studies of Tsoutsoura (2004), Thorne et al. (2014), Lin et al. (2015), Rhou et al. (2016), Wang and Shenghua (2016), and Yang and Suvd (2017). Sweeney (2009) also found a positive association between CSR and FP in SMEs of Ireland. Financial sustainability and social responsibilities were also tested in MFIs of India, Pakistan, Nigeria, and Bangladesh, and a positive relationship was found in prior studies.

Hypothesis 2 shows the association of Corporate Social Responsibility with Operational Self-Sustainability in the microfinance sector of Pakistan. CSR increases operational costs, on the one hand, and the other, enhances the companies' financial performance. The results of the prior studies depict a positive connotation between CSR and FP. The findings of the current study are consistent with previous studies. Hypothesis 3 illustrates the relationship between OSS and Financial sufficiency in the Microfinance sector of Pakistan. OSS was strongly positively associated with Financial sustainability in many prior studies both in developed and developing economies (Adongo and Stork, 2006; Rai et al., 2010; Schäfer and Fukasawa, 2011; Rai and Rai, 2012; Beg, 2016; Esampally and Joshi, 2016; Naz et al., 2019). The current study results are consistent with prior studies except for Cochran and Wood (1984), where a weak correlation was found between CSR-FP. It means that if the firm is operationally sustainable, it is financially self-sufficient. The current study also found a positive correlation between CSR and financial performance—consistent with prior studies.

Hypothesis 4 shows that Operational Self-Sustainability mediates the relationship of CSR and Financial Sustainability in MFIs operating in Pakistan. The results of the study show the partial mediation of OSS on the relationship between CSR and FS. Operational self-sufficiency was not tested as a mediator in prior studies but results depicted the importance of OSS in the model.

### Theoretical Contributions

Stakeholder theory rests on the concept of protection to all firms' stakeholders, not only to shareholders. Stakeholder theory suggests the importance of employees, customers, and society. However, the stakeholder theory has not tested in the MFIs of Pakistan before. MFIs have to trade-off between CSR and financial sustainability; therefore, this study will contribute to existing literature to balance stakeholders' interests (Marrewijk, 2003). The current research will also contribute to signaling theory that will provide signals to stakeholders for making a potential investment (Watts and Zimmerman, 1978) in leading MFIs. Signaling theory is vital only when the financial market is efficient, representing full market information (Watts and Zimmerman, 1978; Fama, 1991).

### Practical Implications

The practical implication of this research is that prior studies (Cochran and Wood, 1984; Brammer and Pavalin, 2006; Brammer et al., 2007; Ofori and Hinson, 2007; Brammer and Millington, 2008; Sweeney, 2009; Sim and Prabhu, 2014; Meyer, 2019) did not reach a consensus on the relationship of CSR and FP. Most of the prior studies found a positive association between CSR and FP, which was already being implemented in the firms. MFIs will do CSR activities, improving their operational sustainability, and ultimately leading to financial sustainability. This research tests the direct relationship between CSR and FP, and the impact of operational self-sufficiency as the mediating variable is included. The mediating role of OSS will further enhance the understanding of the CSR-FP relationship.

The results of the current study, consistent with prior studies, also mention that CSR activities would increase employees' commitment and engagement (Brammer et al., 2007; Sweeney, 2009), customer loyalty (Brammer and Millington, 2008; Lee and Heo, 2009; Sweeney, 2009), increase enterprise reputation (Brammer and Pavalin, 2006; Sweeney, 2009), and enhance accessible social capital (Sweeney, 2009). Therefore, managers are known to implement these CSR activities to obtain those benefits.

### Limitations and Future Research Directions

The study was conducted in MFIs under immense pressure to gain self-sustainability and market value for further investment and outreach. Limited data was collected from managers of microfinance institutions only, which can cause issues for generalizability. Other studies should be conducted to compare CSR activities on financial performance (FP) in MFIs and conventional banks. A comparison of the findings generated from primary data through the questionnaire and secondary data (content analysis of financial statements) should be made to find the best method of conducting this type of study. Corporate governance also plays a vital role in implementing CSR activities and the improvement of financial performance. Hence, corporate governance should be used as a moderating variable to obtain the validity of results.

## Data Availability Statement

The raw data supporting the conclusions of this article will be made available by the authors, without undue reservation.

## Ethics Statement

The studies involving human participants were reviewed and approved by the ethics committee of the Department of Management Sciences, University of Okara, Pakistan. The participants provided their written informed consent to participate in this study.

## Author Contributions

RH, SB, and SH conceived of the presented idea. RH developed the theory and SH performed the computations. SB verified the analytical methods. RH encouraged SH to investigate CSR and Operational self-sustainability and supervised the findings of this work. All authors discussed the results and contributed to the final manuscript.

## Conflict of Interest

The authors declare that the research was conducted in the absence of any commercial or financial relationships that could be construed as a potential conflict of interest.
