# Supplementary material for: Financial Sustainability and Corporate Social Responsibility Under Mediating Effect of Operational Self-Sustainability
Source: Front Psychol. 2020 Dec 14;11:550029. doi: 10.3389/fpsyg.2020.550029 (PMC7793798; doi:10.3389/fpsyg.2020.550029)
Supplement: Supplementary file 1 [file Table_1.docx]

**Appendix**

| **Measurement items** | | **Factor loading** |
| --- | --- | --- |
| ***Customer Retention*** | |  |
| CR_1 | Please estimate the percentage of sales in 2006 that were from repeat customers | 0.825 |
| CR_2 | Please estimate the percentage of new sales in 2006 that came about as a result of recommendations from your current customers. | 0.820 |
| CR_3 | Please estimate the percentage of current customers you would describe as loyal customers (have a positive attitude about the company, recommend the firm/products to others and make repeat purchases). | 0.871 |
| CR_4 | Please indicate the impact of the CSR activities of the firm on customer loyalty. | 0.792 |
| CR_5 | Please indicate the extent to which your organization has improved customer attraction (sales). | 0.848 |
| CR_6 | Please indicate the extent to which your organization has improved customer loyalty (sales). | 0.721 |
| ***Employee Attraction and Loyalty*** | |  |
| EL_1 | Please indicate the extent to which you agree or disagree with the following statement “The firm finds it easy to attract new recruits”. | 0.865 |
| EL_2 | Please indicate the impact of the CSR activities of the firm on employee recruitment. | 0.807 |
| EL_3 | Please indicate the impact of the CSR activities of the firm on employee retention. | 0.868 |
| EL_4 | Please indicate the impact of the CSR activities of the firm on employee motivation. | 0.864 |
| ***Enterprise Reputation*** | |  |
| Rep_1 | Please indicate the rating you believe your customers would give your firm on the basis of staff, quality, environment and community responsibility. | 0.869 |
| Rep_2 | Please indicate the rating you believe your employees would give your firm on the basis of quality of product and services and quality of staff. | 0.847 |
| Rep_3 | Please indicate the rating you believe your employees would give your firm on the basis of environment and community responsibility. | 0.825 |
| Rep_4 | Please indicate the rating you believe other firms in your sector would give your firm on the basis of long term investment value, fair treatment of staff, quality of management. | 0.840 |
| Rep_5 | Please indicate the rating you believe other firms in your sector would give your firm on the basis of environmental responsibility and community responsibility. | 0.905 |
| Rep_6 | Please indicate the impact of the firm’s CSR activities on the reputation of the firm in general. | 0.812 |
| ***Social Capital Availability*** | |  |
| SC_1 | Please indicate the extent to which you agree or disagree with the following statement “This firm easily obtains finance from banks and other lending institutions”. | 0.874 |
| SC_2 | . Please indicate the impact of the CSR activities of this firm on its ability to obtain finance from banks and other lending institutions. | 0.791 |
| SC_3 | Please indicate the extent to which you agree or disagree with the following statement “This firms easily obtains finance from investors”. | 0.813 |
| SC_4 | Please indicate the impact of the CSR activities of this firm on its ability to obtain finance from investors. | 0.780 |
| SC_5 | Please indicate the impact of the CSR activities of this firm on its ability to obtain finance from creditors. | 0.880 |
| ***Operational Self-Sustainability*** | |  |
| OSS_1 | How did the operational self-sustainability of firm in 2019 relate to the previous year? | 0.833 |
| OSS_2 | Please indicate the extent to which your organization has experienced reduction in costs from your CSR activities? | 0.898 |
| OSS_3 | Please indicate how much your firm gain operational self-sufficient from CSR activities? | 0.859 |
| OSS_4 | Please indicate the extent to which you agree or disagree with the following statement “A firm must first be cost/operational sustainable before undertaking CSR”. | 0.888 |
| OSS_5 | Please indicate the impact of the CSR activities of the firm on the financial performance of the firm. | 0.793 |
| ***Financial Sustainability*** | |  |
| FS_1 | How did the net profit of the firm in 2019 relate to the previous year? | 0.845 |
| FS_2 | How did the net sales of the firm in 2019 relate to the previous year? | 0.882 |
| FS_3 | Please indicate the impact of the CSR activities of the firm on the financial performance of the firm. | 0.878 |
| FS_4 | Please indicate the extent to which you agree or disagree with the following statement “A firm must first be profitable before undertaking CSR” | 0.891 |
